# Supplementary material for: Modeling glioblastoma heterogeneity as a dynamic network of cell states
Source: Mol Syst Biol. 2021 Sep 16;17(9):e10105. doi: 10.15252/msb.202010105 (PMC8444284; doi:10.15252/msb.202010105)
Supplement: Supplementary file 6 — Source Data for Figure 5 [file MSB-17-e10105-s004.zip › Figure5A_sourcedata/GSEA_3017/nosigdb_stateB.GseaPreranked.1622795021725/RICHARDS_NOWAKOWSKI_SCIENCE_2017_ASTROCYTE_UPREG.html]

Details for gene set RICHARDS\_NOWAKOWSKI\_SCIENCE\_2017\_ASTROCYTE\_UPREG[GSEA]

|  || Dataset | state43017 |
| Phenotype | NoPhenotypeAvailable |
| Upregulated in class | na\_neg |
| GeneSet | RICHARDS\_NOWAKOWSKI\_SCIENCE\_2017\_ASTROCYTE\_UPREG |
| Enrichment Score (ES) | -0.30393967 |
| Normalized Enrichment Score (NES) | -2.4701855 |
| Nominal p-value | 0.0 |
| FDR q-value | 0.0024813795 |
| FWER p-Value | 0.004 |
Table: GSEA Results Summary

  

Fig 1: Enrichment plot: RICHARDS\_NOWAKOWSKI\_SCIENCE\_2017\_ASTROCYTE\_UPREG      
 Profile of the Running ES Score & Positions of GeneSet Members on the Rank Ordered List

  

| PROBE | GENE SYMBOL | GENE\_TITLE | RANK IN GENE LIST | RANK METRIC SCORE | RUNNING ES | CORE ENRICHMENT || 1 | LRRN3 |  |  | 25 | 0.704 | -0.0020 | No |
| 2 | IGFBP5 |  |  | 67 | 0.590 | -0.0323 | No |
| 3 | PMP22 |  |  | 70 | 0.576 | -0.0077 | No |
| 4 | GFAP |  |  | 132 | 0.487 | -0.0712 | No |
| 5 | CYR61 |  |  | 145 | 0.465 | -0.0661 | No |
| 6 | LYPD1 |  |  | 151 | 0.459 | -0.0514 | No |
| 7 | GPC6 |  |  | 159 | 0.454 | -0.0398 | No |
| 8 | NTM |  |  | 209 | 0.409 | -0.0899 | No |
| 9 | CXXC5 |  |  | 210 | 0.408 | -0.0705 | No |
| 10 | AXL |  |  | 213 | 0.407 | -0.0540 | No |
| 11 | SLC1A3 |  |  | 237 | 0.388 | -0.0682 | No |
| 12 | HEY1 |  |  | 253 | 0.376 | -0.0716 | No |
| 13 | ZFP36L2 |  |  | 279 | 0.358 | -0.0901 | No |
| 14 | DDAH1 |  |  | 300 | 0.346 | -0.1021 | No |
| 15 | PHGDH |  |  | 344 | 0.328 | -0.1476 | No |
| 16 | GRIA4 |  |  | 358 | 0.322 | -0.1507 | No |
| 17 | TACC1 |  |  | 359 | 0.322 | -0.1354 | No |
| 18 | SLC35F1 |  |  | 386 | 0.312 | -0.1575 | No |
| 19 | TGFB2 |  |  | 388 | 0.312 | -0.1441 | No |
| 20 | ID1 |  |  | 390 | 0.311 | -0.1308 | No |
| 21 | IFI27L1 |  |  | 400 | 0.308 | -0.1289 | No |
| 22 | FABP7 |  |  | 414 | 0.302 | -0.1330 | No |
| 23 | SMC5 |  |  | 420 | 0.300 | -0.1259 | No |
| 24 | MEST |  |  | 441 | 0.294 | -0.1403 | No |
| 25 | CHPT1 |  |  | 445 | 0.294 | -0.1306 | No |
| 26 | TTYH1 |  |  | 469 | 0.287 | -0.1496 | No |
| 27 | PLOD2 |  |  | 474 | 0.286 | -0.1416 | No |
| 28 | OLFM2 |  |  | 481 | 0.285 | -0.1366 | No |
| 29 | WWC2 |  |  | 521 | 0.278 | -0.1788 | No |
| 30 | CNN3 |  |  | 527 | 0.276 | -0.1728 | No |
| 31 | GNG12 |  |  | 587 | 0.267 | -0.2439 | No |
| 32 | PAX6 |  |  | 617 | 0.261 | -0.2727 | No |
| 33 | SPATS2L |  |  | 620 | 0.260 | -0.2632 | No |
| 34 | NACC2 |  |  | 624 | 0.259 | -0.2552 | No |
| 35 | SCCPDH |  |  | 638 | 0.257 | -0.2614 | No |
| 36 | NLGN4X |  |  | 641 | 0.257 | -0.2520 | No |
| 37 | MCC |  |  | 664 | 0.253 | -0.2713 | No |
| 38 | SCD |  |  | 688 | -0.263 | -0.2914 | Yes |
| 39 | MSMO1 |  |  | 691 | -0.267 | -0.2816 | Yes |
| 40 | VIM |  |  | 692 | -0.267 | -0.2689 | Yes |
| 41 | MMP16 |  |  | 707 | -0.285 | -0.2752 | Yes |
| 42 | ID4 |  |  | 708 | -0.285 | -0.2617 | Yes |
| 43 | DSEL |  |  | 709 | -0.297 | -0.2476 | Yes |
| 44 | MYO10 |  |  | 710 | -0.305 | -0.2331 | Yes |
| 45 | PCDH10 |  |  | 713 | -0.305 | -0.2214 | Yes |
| 46 | NEAT1 |  |  | 714 | -0.308 | -0.2067 | Yes |
| 47 | ZEB1 |  |  | 717 | -0.317 | -0.1945 | Yes |
| 48 | PRDX1 |  |  | 719 | -0.326 | -0.1804 | Yes |
| 49 | FGFBP3 |  |  | 721 | -0.334 | -0.1660 | Yes |
| 50 | OLIG1 |  |  | 729 | -0.370 | -0.1583 | Yes |
| 51 | IDI1 |  |  | 734 | -0.397 | -0.1451 | Yes |
| 52 | HMGCS1 |  |  | 739 | -0.418 | -0.1309 | Yes |
| 53 | DBI |  |  | 740 | -0.422 | -0.1108 | Yes |
| 54 | CREM |  |  | 750 | -0.496 | -0.1001 | Yes |
| 55 | PDGFRA |  |  | 758 | -0.673 | -0.0780 | Yes |
| 56 | FABP5 |  |  | 759 | -1.641 | -0.0000 | Yes |
Table: GSEA details [plain text format]

  

Fig 2: RICHARDS\_NOWAKOWSKI\_SCIENCE\_2017\_ASTROCYTE\_UPREG: Random ES distribution      
 Gene set null distribution of ES for **RICHARDS\_NOWAKOWSKI\_SCIENCE\_2017\_ASTROCYTE\_UPREG**
